# The Effect of Age on Osteogenic and Adipogenic Differentiation Potential of Human Adipose Derived Stromal Stem Cells (hASCs) and the Impact of Stress Factors in the Course of the Differentiation Process

**DOI:** 10.1155/2015/309169

**Published:** 2015-07-12

**Authors:** Katarzyna Kornicka, Krzysztof Marycz, Krzysztof Andrzej Tomaszewski, Monika Marędziak, Agnieszka Śmieszek

**Affiliations:** ^1^Electron Microscopy Laboratory, Wroclaw University of Environmental and Life Sciences, 50-631 Wroclaw, Poland; ^2^Wroclaw Research Centre EIT+, 54-066 Wroclaw, Poland; ^3^Department of Anatomy, Jagiellonian University Medical College, 31-034 Krakow, Poland; ^4^Department of Animal Physiology and Biostructure, Faculty of Veterinary Medicine, Wroclaw University of Environmental and Life Sciences, 50-375 Wroclaw, Poland

## Abstract

Human adipose tissue is a great source of autologous mesenchymal stem cells (hASCs), which are recognized for their vast therapeutic applications. Their ability to self-renew and differentiate into several lineages makes them a promising tool for cell-based therapies in different types of degenerative diseases. Thus it is crucial to evaluate age-related changes in hASCs, as the elderly are a group that will benefit most from their considerable potential. In this study we investigated the effect of donor age on growth kinetics, cellular senescence marker levels, and osteogenic and adipogenic potential of hASCs. It also has been known that, during life, organisms accumulate oxidative damage that negatively affects cell metabolism. Taking this into consideration, we evaluated the levels of nitric oxide, reactive oxygen species, and superoxide dismutase activity. We observed that ROS and NO increase with aging, while SOD activity is significantly reduced. Moreover cells obtained from older patients displayed senescence associated features, for example, *β*-galactosidase activity, enlarged morphology, and p53 protein upregulation. All of those characteristics seem to contribute to decreased proliferation potential of those cells. Our results suggest that due to aging some cellular modification may be required before applying aged cells efficiently in therapies such as tissue engineering and regenerative medicine.

## 1. Introduction

One of the currently fastest developing fields of medical science is regenerative medicine. This results from the need for advanced treatment options for many diseases, which are becoming more frequent due to the aging of society. The main target populations that may most benefit from advances in regenerative medicine are elderly patients. In recent years, many basic researchers as well as clinical groups showed the positive effect of mesenchymal stem cells (MSCs) as therapeutic agents, both* in vivo* and* in vitro* [1, 2]. MSCs, in general, are characterized as a source of cells that possess a unique proliferative potential, the ability to self-renew, and can also differentiate* in vitro* into multiple lineages [3–5]. Besides proliferative and differentiation potential, MSCs are well known for their immunomodulatory and immunosuppressive properties, which make them an even more promising tool in regenerative medicine [6]. In a wide range of animal model studies [7], it has been demonstrated that MSCs can be successfully applied in regeneration of musculoskeletal tissues [8], cardiac tissues [9], and neurological disorders [10]. One of the potential explanations of the regenerative ability of MSCs is paracrine action through secretion of membrane derived vesicles (MVs) which contain a wide range of growth factors, as well as antiapoptotic and anti-inflammatory factors [11]. The most often investigated and described in literature MSC populations are those isolated from bone marrow (bone marrow derived mesenchymal stem cells (BMSC)) and those of adipose origin (human adipose derived mesenchymal stem cells (hASC)). However, BMSC application is limited, because of the rather complicated acquisition process, requirement of using extensive anesthesia, and finally low cell yield [12]. Compared with BMSCs, hASCs have distinct advantages in cell preparation due to ease and safety of adipose tissue obtaining. Human ASCs, like BMSCs, are characterized by similar phenotype, stability over long term culture, and extended efficiency* in vitro* [13]. Moreover, in our previous study we have shown that hASCs, in comparison to BMSCs, have greater potential for osteogenic differentiation when cultured onto metallic biomaterials [14]. Thereby, consideration of hASCs' application in clinical practice seems to be more real in the shorter perspective. Because of the growing interest in stem cell-based therapy and tissue engineering there are some points which should be addressed before their clinical application, especially in the context of elderly donors. It was demonstrated that donor age negatively correlates with the number and proliferative potential of BMSCs, at the same time positively correlating with the level of cell apoptosis and senescence [15].

Recently, a growing number of factors are being analyzed in order to investigate their role in MSCs aging and their potential input in MSCs proliferative and differentiative status [16]. Oxidative stress, as an imbalance between free radicals and antioxidants, has been reported to influence MSCs multipotent character on the level of particular gene expression, proliferation, and differentiation. Oxidative stress factors including reactive oxygen species (ROS) and nitric oxygen (NO) are implicated in cellular proliferation and differentiation capacity [17]. They are also involved in initiating apoptosis, which is characterized by upregulation of the p53 gene (tumor suppressor), changes in the expression of pro- and antiapoptotic Bcl-2 family members, cytochrome C relocation, activation of caspases, chromatin condensation, and DNA fragmentation [18]. On the other hand, the activity of free radicals scavengers, like superoxide dismutase (SOD), that balance the effects of free radicals might have a crucial role in evaluation of MSCs both proliferative and differentiative status in the context of donor age.

Recently, there is a growing interest in the application of hASCs in clinical practice and tissue engineering. Thus the evaluation of their cytophysiological features in the context of donor age seems to be necessary. Bearing in mind the fact that the regenerative potential of MSCs is linked to their proliferative, differentiative, and paracrine activity, in this study we evaluated hASCs morphology, growth kinetics, and osteogenic* versus* adipogenic differentiation potential of cells obtained from donors of different age. We performed our study in regard to gene expression profile, correlation of ROS/NO* versus* SOD activity, and senescence level. 

## 2. Materials and Methods

### 2.1. Cell Collection and Isolation

The protocol was approved by the Local Bioethics Committee of Wroclaw Medical School (registry number KB-177/2014). All donors had given their written informed consent prior to the procedure. The study has been performed in accordance with the ethical standards laid down in the 1964 Declaration of Helsinki and its later amendments.

Human subcutaneous adipose tissue was collected from both male and female subjects, age range 23–77 years (54.3 ± 21.9) during total hip arthroplasty. Patients were divided into 4 age groups: >20 (age range 20–29, *n* = 7, mean age 24 ± 1.4 years), >50 (age range 50–60, *n* = 7, mean age 57.5 ± 0.7), >60 (age range 60–69, *n* = 7, age 67), and >70 (age range 70–79, *n* = 7, mean age 75 ± 2.8).

All adipose tissue samples were processed under the same conditions. After surgical harvesting adipose tissue samples were placed in Hank's Balanced Salt Solution (HBSS, Sigma Aldrich). Adipose derived stem cells' isolation was performed according to a previously described protocol by Grzesiak et al. [19] and Marycz et al. [20], under sterile conditions. Briefly, the tissue was washed extensively with HBSS supplemented with 1% antibiotic-antimycotic solution (penicillin/streptomycin/amphotericin B solution, Sigma Aldrich) and then cut into small pieces using surgical scissors. The extracellular matrix was digested with 1 mg/mL collagenase type I (Sigma Aldrich) and incubated for 40 minutes at 37°C. Then the suspension was centrifuged at 1200 g for 10 minutes. After removing the supernatant, the pellet was resuspended in the culture medium and transferred to a culture flask. Primary culture of hASCs' was designated as “passage 0.” To prepare cells for experiment, they were passaged three times.

### 2.2. Immunophenotyping

Human ASCs were recognized by immunophenotyping using fluorochrome conjugated monoclonal antibodies specific for CD29, CD34, CD45, CD90, CD73b, CD44, and CD105 (all antibodies purchased from BD Pharmingen). Isotype-matched antibodies were used as controls. Due to immunophenotyping hASCs from passage 1 were detached using TrypLE Express solution (Life Technologies), washed with phosphate buffered saline (PBS, Sigma Aldrich) containing 2% FBS, and resuspended at total of 5*∗*10^5^ cells/mL. Cell suspension was incubated at 4°C for 20 min with the specific antibodies preconjugated with allophycocyanin (APC), peridinin chlorophyll protein complex (PerCP), fluorescein isothiocyanate (FITC), or phycoerythrin (PE). At least ten thousand stained cells were acquired and analyzed by Becton Dickinson FACS Calibur flow cytometer. The samples were analyzed using FlowJo software (TreeStar).

### 2.3. Cell Culture

During the experiment, cells were cultured under aseptic and unchanging conditions in an incubator (37°C, 5% CO2, and 95% humidity). Primary cultures were plated on T-25 flasks in culture media containing Dulbecco's Modified Eagle's Medium (DMEM, Sigma Aldrich) with nutrient F-12 Ham, 10% of Fetal Bovine Serum (FBS, Sigma Aldrich), and 1% solution of P/S/A. The media were changed every second day. Passage was performed when cells reached about 80% confluence. Human ACSs culture passaging was performed using TrypLE Express solution according to manufacturer protocol. Passaged cultures were denoted as passage 1.

### 2.4. Colony Forming Unit-Fibroblastic Assay (CFU-F), Cell Proliferation Assay, and hASC Morphology Evaluation

To evaluate the ability to form colonies, the CFU-F assay was performed as described previously with modifications [21]. Briefly, cells were plated on six-well culture plates at the appropriate density (1*∗*10^3^ cells/well) in culture media (DMEM, 10% FBS, 1% P/S/A). The medium was changed every two days and cultures were maintained for 7 days. After fixation in 4% ice-cold paraformaldehyde, cells were stained with pararosaniline (Sigma Aldrich) and colonies of more than 50 cells were scored. The efficiency of colony forming (CFE) ability was calculated using the formula mentioned below, as presented elsewhere [22]

(1) $$\begin{array}{c} \text{CFE}\left[\;\%\;\right]=\left(\;\frac{\text{number of colonies}}{\text{initial cell number}}\;\right)\cdot 100\%. \end{array}$$

Cell proliferation rate was evaluated using 10% resazurin-based dye-TOX-8 (Sigma Aldrich) following manufacturer's protocols. To perform the assay, media were collected and replaced with the culture medium containing 10% resazurin dye solution. Then cultures were incubated with dye in a CO_2_ incubator, 37°C for 2 hours. Next, the supernatants were collected and transferred to a 96-well plate to perform the spectrophotometric assay (BMG Labtech, Germany). The absorbance was measured at a wavelength of 600 nm for resazurin and 690 nm reference wavelength. Each test included a blank (dye containing medium without cells) for control. The test was performed at the 2nd, 5th, 7th, 12th, 14th, and 16th day of the experiment. Cell number was obtained from test data. Population doubling time was calculated using online software [23].

Cells' morphology (induced and noninduced) was evaluated using an epifluorescent microscope (Zeiss, Axio Observer A.1) and a scanning electron microscope (SEM, Zeiss Evo LS 15). Morphology analysis was performed on the 7th (noninduced cells), 14th day (cells from adipogenic differentiation), and 16th day (cells from osteogenic differentiation). Prior to fluorescent microscopy observations samples were first (a) rinsed three times with HBSS; (b) fixed with 4% paraformaldehyde for 45 min; (c) washed again (as described above); (d) permeabilized for 15 min with 0.1% Triton X-100 at room temperature and washed again. Actin filaments were stained using atto-488-labeled phalloidin (Sigma Aldrich) at dilution 1 : 800 with HBSS for 40 minutes in the dark at room temperature and cells' nuclei were counterstained with diamidino-2-phenylindole (DAPI; 1 : 1000, Sigma Aldrich) for 5 minutes. Mitochondria were stained intravitally using Mito Red fluorescence dye (Sigma Aldrich) following manufacturer's protocol. Tubulin was labeled with red fluorescent protein in live cells with CellLight Tubulin-RFP, BacMam 2.0 (Life Technologies), and the Golgi apparatus was stained green with CellLight Golgi-GFP, BacMam 2.0 (Life Technologies). Cells' nuclei were counterstained with DAPI. All procedures were performed in accordance with manufacturer's protocols. Images were taken using Canon PowerShot Camera.

### 2.5. Oxidative Stress Factors and Senescence Analysis in hASCs Culture

To evaluate stress levels, cells were cultured in normal growth media without phenol red for two days. Nitric oxide concentration was assessed using commercially available Griess reagent kit (Life Technologies). Superoxide dismutase (SOD) activity was measured using a SOD Assay kit (Sigma Aldrich). Reactive oxygen species (ROS) were estimated by incubating cells with an H2DCF-DA (Life Technologies) solution. All procedures were performed according to manufacturer's protocols.

To identify the presence of senescence associated *β*-galactosidase (*β*-gal), cells were stained using a commercial kit (Senescence Cells Histochemical Staining Kit, Sigma Aldrich) according to manufacturer's protocol. Supernatant was then collected and followed by measurement of absorbance. Cells were then observed under an inverted microscope (Zeiss, Axio Observer A.1) and percentage of cells expressing *β*-gal (stained blue) in regard to *β*-gal negative cells was calculated.

Amount of viable and dead cells was evaluated with Cellstain Double Staining Kit (Sigma Aldrich). Viable cells were stained with Calcein-AM and emitted green fluorescence, whereas dead cells' nuclei were stained orange with Propidium Iodide. Cells were then observed using fluorescence microscopy (Zeiss, Axio Observer A.1). The percentage of dead cells was calculated. All procedures were performed in accordance with manufacturer's instructions.

Staining for caspase-3 was performed using anti-caspase-3 antibodies (anti-caspase-3-active antibody produced in rabbit, Sigma Aldrich, category number C8487).

### 2.6. Multipotency Assay: Oil Red O, Alizarin Red Staining, SEM, and SEM-EDX Analysis

To identify the multilineage differentiation potential of hASCs, a multipotency test was performed using commercially available differentiation media (STEMPRO Osteogenesis Differentiation Kit and STEMPRO Adipogenesis Differentiation Kit, both Life Technologies). The cells were seeded onto 24-well dishes at a concentration 2*∗*10^4^ cells per well. Stimulation of adipogenesis lasted 14 days, whereas osteogenesis lasted 16 days. All procedures were performed according to manufacturer's instructions. Media were changed every three days. Cultures expanded in standard growth medium were used as a control to allow for establishing differentiation effectiveness. Multilineage differentiation was confirmed at 2 weeks after induction by cells staining. Multipotency test was performed on ASCs isolated from patients between 25 and 77 age ranges.

To identify intracellular lipid vacuoles and confirm adipogenesis, the Oil Red O (Sigma Aldrich) staining was performed. Media were removed and the cells were fixed in 4% paraformaldehyde at room temperature for 45 min, followed by incubation with 60% isopropanol for 5 min. Slides were then incubated with Oil-Red O for 15 minutes. The cells were also counterstained with haematoxylin for 1 min. Excessive dye was washed away with PBS. The Oil Red O-stained areas, as a marker of lipid accumulation, were analyzed using Image J software (NIH). In each group, the mean for five independent sections was used for the analysis.

To visualize extracellular matrix mineralization, cells were fixed with 4% paraformaldehyde at room temperature for 10 min. After washing with distilled water, 2% Alizarin Red solution was added to wells. After 10 min the dye was removed and cells were washed with distilled water 3 times. Cells were observed under an inverted microscope (AxioObserverA1, Zeiss) and pictures were taken using Canon PowerShot digital camera.

Detailed cell morphology was evaluated using SEM (EVO LS15, Zeiss). In order to perform observations cells were rinsed with distilled water and dehydrated in graded ethanol series (concentrations from 50% to 100%). Dehydrated samples were then sputtered with gold (ScanCoat 6, Oxford), placed in a microscope chamber, and observed using the SE1 detector, at 10 kV of filament's tension. Additionally calcium and phosphorus concentration was analyzed by SEM with energy dispersive X-ray analysis (SEM/EDX). The quantax detector (Brüker) with 10 kV of filament tension was used to perform a line scan analysis of randomly selected cells. The obtained values were presented as weight percentage (wt%). Moreover, using SEM/EDX technique, we estimated number and size of bone nodules in each age group.

### 2.7. Quantitative Osteopontin, Osteocalcin, Collagen Type I, Leptin, Adiponectin, and Alkaline Phosphatase Activity Assay

To examine extracellular protein levels, supernatants of cultures were collected and analyzed by enzyme-linked immunosorbent assay (ELISA). The presence of osteopontin (OPN) and collagen type 1 (Col-1) was determined on the 16th day using Human OPN Quantikine ELISA Kit (R&D Systems, Abingdon, UK) and Human Pro-Collagen I alpha 1 DuoSet (R&D Systems, Abingdon, UK), respectively. To evaluate the amount of adiponectin and leptin supernatants were collected on the 14th day and an ELISA assay was performed (Wuhan EIAab Science Co., China). Analysis was performed in accordance with manufacturers' protocols, and protein amount was expressed as ng/mL of supernatant.

To estimate extracellular alkaline phosphatase (ALP) activity, supernatants were collected at 2nd, 7th, 11th, 14th, and 16th day. Assay was performed using an Alkaline Phosphatase Colorimetric Assay Kit (Abcam, Cambridge, UK) according to manufacturer's protocol. Briefly, in the assay, the p-nitrophenyl phosphate (pNPP) was used as a phosphatase substrate. The substrate was hydrolyzed into p-nitrophenol by ALP. The product was then measured spectroscopically at 405 nm wavelength (BMG Labtech, Germany). The amount of pNP was obtained by sample readings applied to a standard curve. ALP activity was calculated using the following formula: ALP activity (U/mL) = *A*/*V*/*T* (where *A* is pNP amount; *V* is volume of sample added to well (mL); *T* is reaction time)

### 2.8. Quantitative Real-Time Reverse Transcription Polymerase Chain Reaction (qRT-PCR) for p53, Col-1, OPN, BMP-2, ALP, OCN, and PPAR*γ*

After adipogenic and osteogenic stimulation cells were rinsed using HBSS followed by homogenization in TRI Reagent (Sigma Aldrich). Total RNA was isolated using the phenol–chloroform method as previously described by Chomczynski and Sacchi [24].

DNA-free RNA was prepared using DNase I RNase-free kit (Thermo Scientific). Five-hundred ng of total RNA was used for each reaction. Transcription of gDNA-free total RNA was reverse transcribed to cDNA using oligo(dT)18 primers and Moloney Murine Leukemia Virus Reverse Transcriptase (M-MLV RT, Novazym). qRT-PCRs were carried out on a CFX Connect Real-Time PCR Detection System (BioRad) containing 2 *μ*L of cDNA in a total volume of 20 *μ*L, 500 nM primers, and the SensiFast SYBR & Fluorescein Kit (Bioline). The sequences of the primers used for amplification of the gene are listed in Table 1. Relative gene expression analysis (Qn) was calculated in relation to the housekeeping gene GAPDH.

### 2.9. Statistical Analysis

Group data were presented as bar chart (mean ± SD) or box pot (Whisker's from min to max values) of at least 2 independent experiments (biological replicates, *n* ≥ 2) measured as duplicate or more (technical replicates, *n* ≥ 2). Statistical significance was determined using one-way analysis of variance (ANOVA) with Tukey's post hoc multiple comparison test or two-way analysis of variance (two-way ANOVA) (Prism5.04, GraphPad Software, CA, USA). *P* < 0.05 was considered statistically significant.

## 3. Results

### 3.1. Immunophenotyping and Multipotency Assay

**Figure 1:**
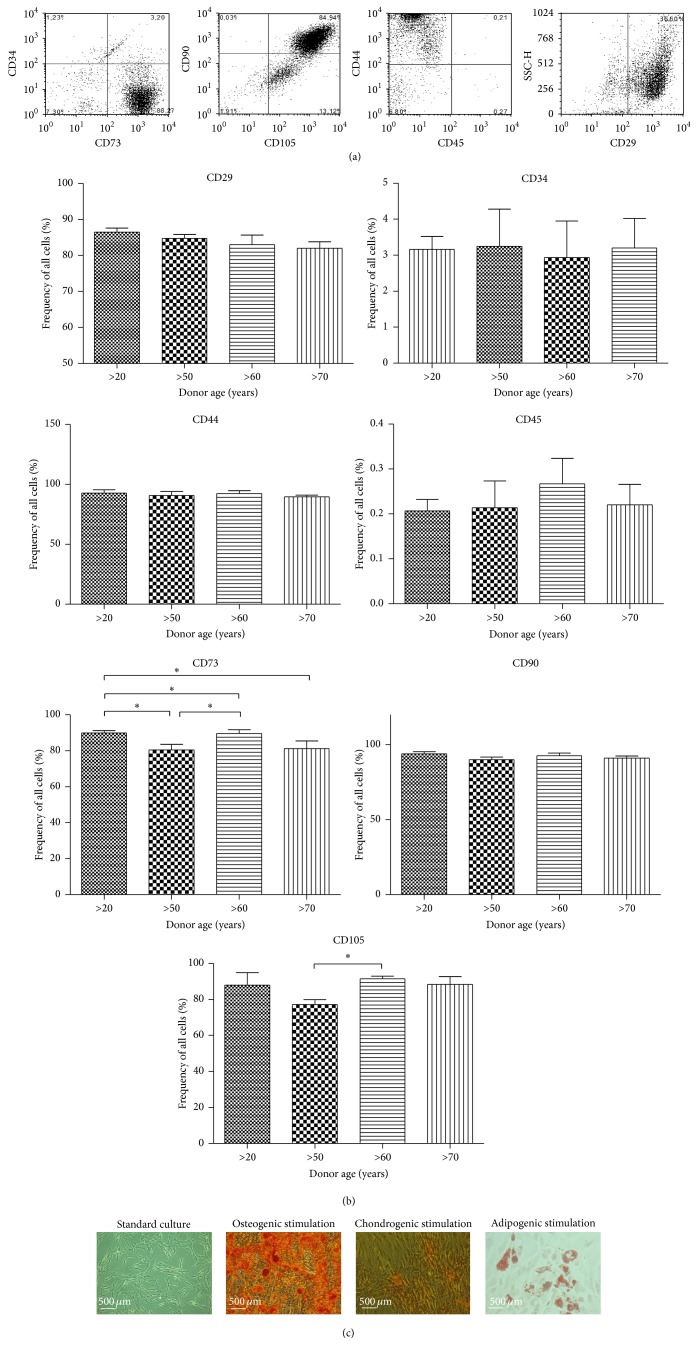
Flow cytometry and multipotency analysis. Flow cytometry dot plots representative for human adipose derived mesenchymal stem cells (hASCs) (a). Isolated cells are characterized by the presence of CD29, CD44, CD73b, CD90, and CD105. hASCs lack the CD45 surface markers, as well as the CD34hematopoietic lineage marker. Flow cytometry analysis determined the percentage of specific markers in total analyzed hASCs from patients in different age (b). Quantification of markers revealed no significant differences between groups. Representative images from trilineage assay of hASCs after 21 days of differentiation (c). Osteogenically differentiated cells in monolayer stained with Alizarin Red, chondrogenically differentiated cells using Safranin O, and adipogenically differentiated cells stained using Oil Red O. Multipotency test was performed on ASCs isolated from patients between 25 and 77 age ranges. Magnification ×100, scale bars: 500 *μ*m. Results expressed as mean ± SD. ^*∗*^ *P* value < 0.05.

Immunophenotype characterization of hASCs using flow cytometry revealed that cells expressed the following markers, CD44, CD73b, CD90, and CD105, but did not express the CD34 hematopoetic lineage marker and the leukocyte common antigen CD45 (Figure 1(a)). Percentage of cells expressing specific antigen was then calculated (Figure 1(b)). We observed an age-related change in expression of CD73 marker between young and older donors (*P* < 0.05). Isolated hASCs differentiated into osteogenic, chondrogenic, and adipogenic lineages, what was verified by means of specific staining (Figure 1(c)).

### 3.2. Effect of Age on hASCs Morphology, Growth Kinetics, and Clonogenic Potential

**Figure 2:**
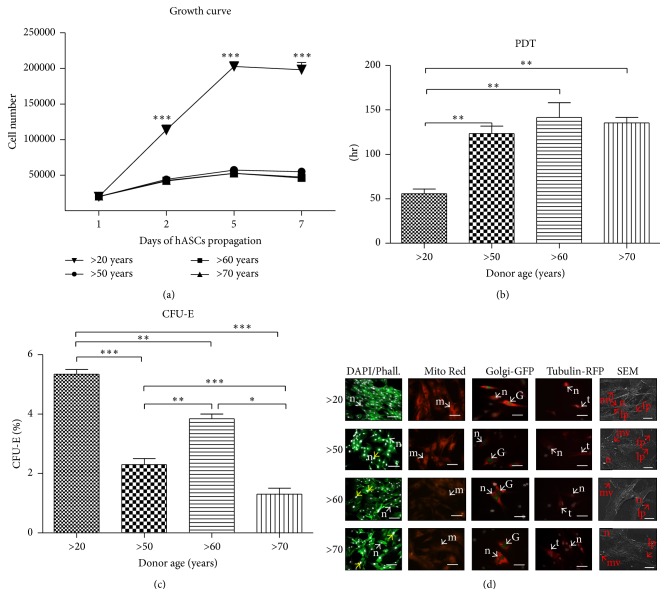
Effect of age on hASCs morphology, growth kinetics, and clonogenic potential. Mean cell number for each age group with respect to culture duration (a). Significant differences in proliferation potential were observed between younger (>20) and older patients (50–70). Cell population doubling time expressed according to donor age (b). CFU-E assay showing percentage of colonies consisting of more than 50 cells between different age groups (c). Morphology and cellular composition of hASCs from different age groups, evaluated after seven days of propagation (d). Typical morphological features were indicated with proper abbreviations: n: nucleus, m: mitochondria, G: Golgi apparatus, t: microtubules, mv: microvesicles, lp: lamellipodia, and fp: filopodia. Enlarged nuclei were pointed out with yellow arrows. Magnification: DAPI/Phalloidin (DAPI/Phall.) ×100, scale bar: 250 *μ*m; Mito Red, Golgi-GFP, Tubulin RFP: ×200, and scale bars 125 *μ*m; SEM: ×2000, scale bars: 30 *μ*m. Results expressed as mean ± SD. ^*∗*^ *P* value < 0.05, ^*∗∗*^ *P* value < 0.01, and ^*∗∗∗*^ *P* value < 0.001; (>20), (>50), (>60), and (>70): donor age range.

To evaluate growth kinetics of hASCs isolated from four age groups, the cells were seeded at the same initial density of 2*∗*10^3^ cells/well (day 1). Cells from younger donors (>20 years of age) displayed a significantly higher proliferation rate in comparison to older patients, whereas no significant change between samples from older donors was observed (Figure 2(a)).

The cells from younger donors (>20 years of age) reached population doubling (PDT) the quickest (55 ± 9.8 h) showing a significant difference (*P* < 0.01) in comparison to older patients. The longest PDT was observed in the >60 years of age group (141.5 ± 23.8 h), showing a 2.7-fold longer PDT; however there were no significant differences between elderly donors (Figure 2(b)).

The greatest number of clonogenic fibroblast precursor cells (CFU-F) was observed in younger donors (>20 years of age). Among elderly donors a significant elevated level of CFU-F was noticed in the >60 years group when compared to the >50 and the >70-year-old donors (Figure 2(c)).

In the initial stage of hASCs culture, cells obtained from young donors displayed the typical, elongated fibroblast-like morphology. Moreover they were concentrated more densely and closely adhere to each other. Staining after 168 h revealed multilayer formation and centrally positioned nuclei (Figure 2(d)). Cells from younger donors exhibited spindle-shaped morphology, whereas cells from >50 and >70 years of age groups exhibited a more flat, extended morphology, being amorphous in shape and no longer bipolar. Some of these cells also displayed larger nuclei size. Culture confluence after 168 h reached approximately 80%.

To evaluate mitochondrial activity, the Mito Red staining was applied. Fluorescence microscopy images showed a strong fluorescent signal in younger donors when compared to other groups (Figure 2(d)). In elderly patients, mitochondria were mainly located around nuclei with a strong fluorescent signal (Figure 2(d)).

The staining for Golgi apparatus revealed comparable fluorescent signal for all investigated groups (Figure 2(d)). The most intensive signal for microtubules' staining was observed in younger donors (Figure 2(d)).

Membrane derived microvesicles (MVs) were observed mainly on the cell border, showing the greatest distribution in the >20 years patients. Morphological variations of shape and amount of lamellipodia were also observed. Cells from the >20 and >60-year-old age groups developed the longest structures which were able to connect neighboring cells. The smallest-scale cytonemes web was found in the >70 years of age group.

### 3.3. Effect of Age on Senescence and Apoptosis

**Figure 3:**
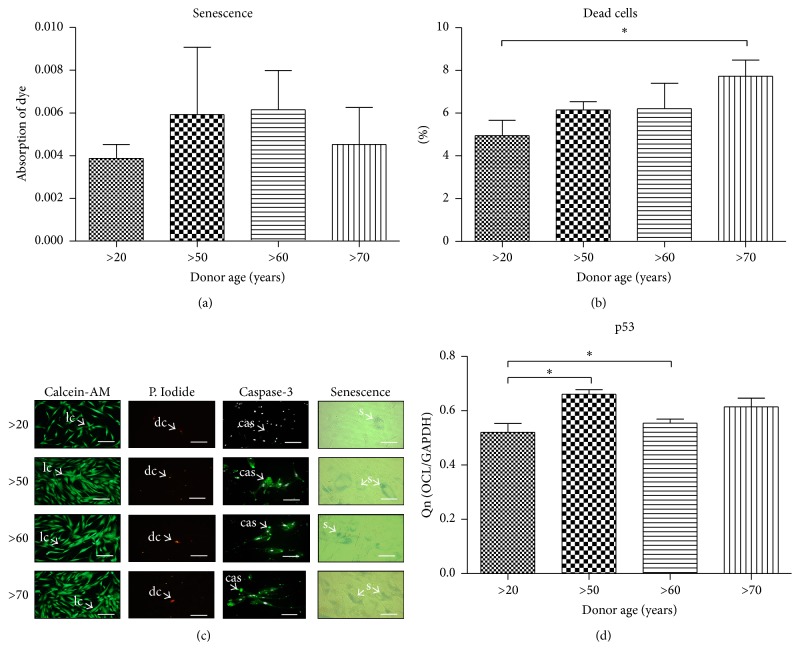
Effect of age on senescence and apoptosis. Quantification of senescence staining showing differences in dye absorption (a). Percentage of dead cells calculated from Calcein-AM/Propidium Iodide staining (b). Cells stained with Calcein-AM, Propidium Iodide, antibodies for caspase-3 (nuclei counterstained with DAPI) and senescence dye showing cells with *β*-galactosidase accumulation (c). Evaluation of p53 mRNA levels (d). Abbreviations used to describe different cell types: lc: live cell, dc: dead cell, cas: caspase-3 positive cell, and sc: *β*-galactosidase positive cell. Magnification ×100, scale bars: 250 *μ*m. Results expressed as mean ± SD. ^*∗*^ *P* value < 0.05. (>20), (>50), (>60), and (>70): donor age range.

We examined qualitative (Figure 3(c)) and quantitative, by amount of dye absorption (Figure 3(a)) *β*-gal positive cells. Obtained data showed an increase *β*-gal production with age. To determine whether the age-related decrease in cell growth results from apoptosis level, we evaluated the percentage of dead cells in culture (Figures 3(b) and 3(c)). The number of dead cells increased with donor age, though without statistical significance (*P* > 0.05). It reached 4.95 ± 1.4% in >20, 6.2 ± 0.8% in >50, 6.2 ± 2% in >60, and 7.7 ± 1.3% in >70 years age group. Caspase-3 fluorescence signal intensity was higher in the >50 and the >70 years of age group in comparison to the >20 and >60 years groups (Figure 3(c)). We have also observed differences between the >20 and >50 years old patients' expression of p53 mRNAs (Figure 3(d)).

### 3.4. Oxidative Stress Factors Analysis in hASCs

**Figure 4:**
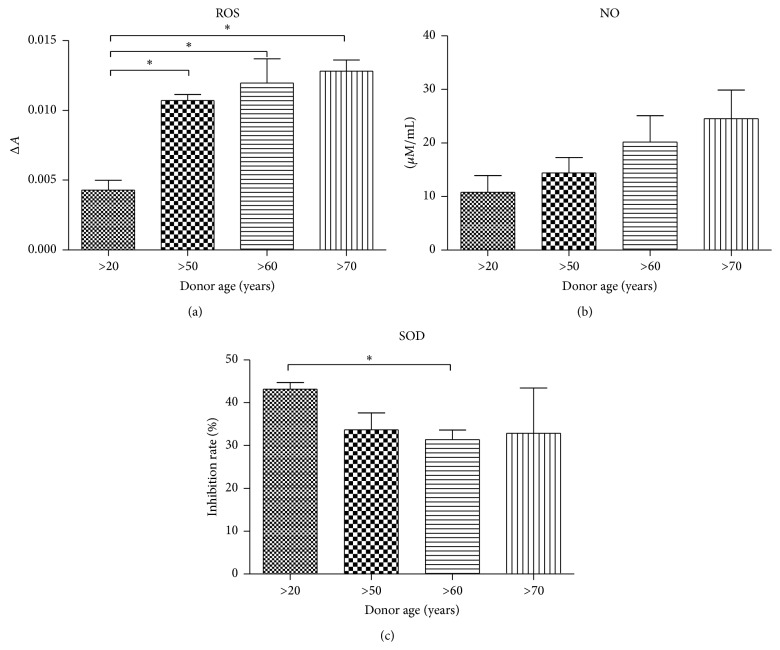
Analysis of oxidative stress factors in hASCs. Reactive oxygen species (a), nitric oxide (b), and superoxide dismutase (c). Results expressed as mean ± SD. ^*∗*^ *P* value < 0.05.

Obtained data showed higher ROS levels in older donors (50–70 years) in comparison to the youngest group (>20), with no significant difference between >50, >60, and >70-year age groups (Figure 4(a)). Similar to ROS in case of NO, progressive increasing of NO amount was positively correlated with donor age (Figure 4(b)). We observed that SOD activity decreased with donor age (Figure 4(c)).

### 3.5. Age-Related Changes in hASC Cultured under Osteogenic Conditions

**Figure 5:**
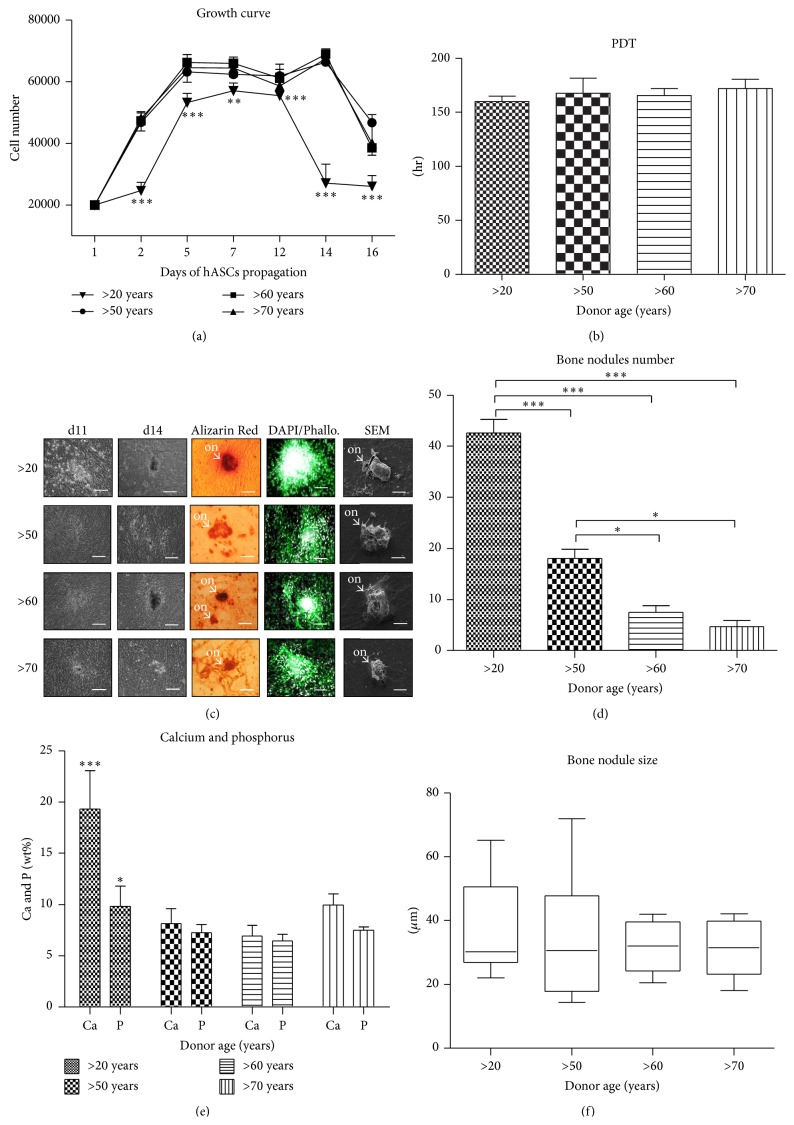
Age-related changes in hASC cultured under osteogenic conditions. Growth curves (a), population doubling time (b). Morphological evaluation after 16 days of culture, magnification ×100, scale bars: 250 *μ*m; SEM pictures; magnification ×2000, scale bars: 20 *μ*m (c). Mean bone nodules number (d), composition of mineral matrix (e), and average nodules diameter (f). Results expressed as mean ± SD. ^*∗*^ *P* value < 0.05, ^*∗∗*^ *P* value < 0.01, and ^*∗∗∗*^ *P* value < 0.001. (>20), (>50), (>60), and (>70): donor age range; d11: day 11th, d14: day 14th, and on: osteo-nodule.

hASCs isolated from all donors were cultured under osteogenic conditions in order to evaluate their osteogenic differentaion potential on a functional level. A significantly lower proliferative activity was observed in osteoblasts precursors (Op) isolated from elderly donors when compared to other groups. There were no significant changes in growth kinetics of Op among elderly indyviduals (Figure 5(a)). Population doubling time (PDT) was comparable between all investigated groups, showing no significant differences between donors of different age (Figure 5(b)).

The level of extracellular matrix calcification was detected using Alizarin Red staining and SEM-EDX technique. The most abundant reaction for Alizarin Red as well as the highest concentration of calcium and phosporus was observed in hASCs of youngest donors (Figure 5(c)). Moreover, the largest number of formed osteonodules was observed in the youngest donors >20 when compared to older groups (Figure 5(d)). Furthermore we explored the relationship between calcium and phosphorus amount in bone nodules. The measurement revealed significant differences between young and elderly donors, showing age-related decrease of calcium and phosphorus deposition (Figure 5(e)) (*P* < 0.001 and *P* < 0.05 for calcium and phosphorus, resp.). However, there were no significant changes between investigated groups in the size of osteonodules formed (Figure 5(f)).

**Figure 6:**
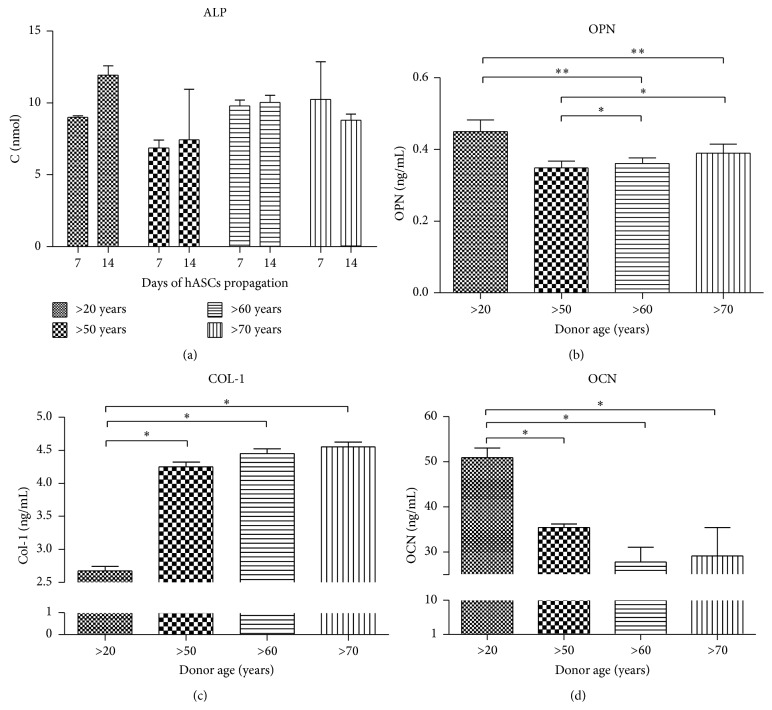
Protein levels in hASCs osteogenic culture. Alkaline phosphatase activity, evaluated on 7th and 14th day of culture in osteogenic conditions (a). Quantitative ELISA analysis of extracellular osteopontin (b), collagen I (c), and osteoclacin (d). Results expressed as mean ± SD. ^*∗*^ *P* value < 0.05, ^*∗∗*^ *P* value < 0.01.

The highest activity of ALP in the last stage of osteogenic culture (14th day) was observed in youngest donors (Figure 6(a)). A similar tendency was noticed with OPN protein levels (Figure 6(b)). Interesiengly a lower concentration of Col-1 protein was observed in younger patients (Figure 6(c)). We observed that OCN amount decreases with age as well (Figure 6(d)).

### 3.6. Analysis of Oxidative Stress Factors in Osteoblast Precursor (Op)

**Figure 7:**
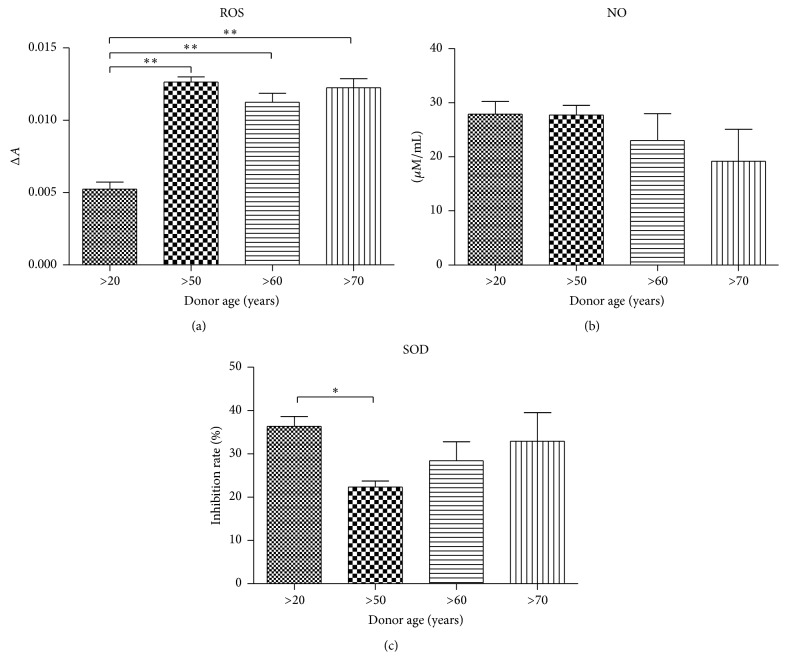
Analysis of oxidative stress factors in osteoblast precursors (Op). Reactive oxygen species (a), nitric oxide (b), and superoxide dismutase (c). Results expressed as mean ± SD. ^*∗*^ *P* value < 0.05, ^*∗∗*^ *P* value < 0.01.

Analysis of oxidative stress level in Op was performed at the last stage of osteogenic culture of hASC. Reactive oxygen species (ROS) levels significantly increased with age, being the lowest in young donors. However, there were no significant changes in ROS concentration between other groups (Figure 7(a)). Nitric oxide levels were elevated in youngest donors when compared to other groups. However, there were no significant changes between donor age groups (Figure 7(b)). Superoxide dismutase (SOD) activity was significantly higher in young Op as compared to aged cells (Figure 7(c)). SOD activity progressively increased in elderly patients.

### 3.7. The Expression of Osteocalcin (OCL), Osteopontin (OPN), Bone Morphogenic Protein 2 (BMP-2), Collagen Type I (Col-1), and Alkaline Phosphatase (ALP) in Age-Related Op

**Figure 8:**
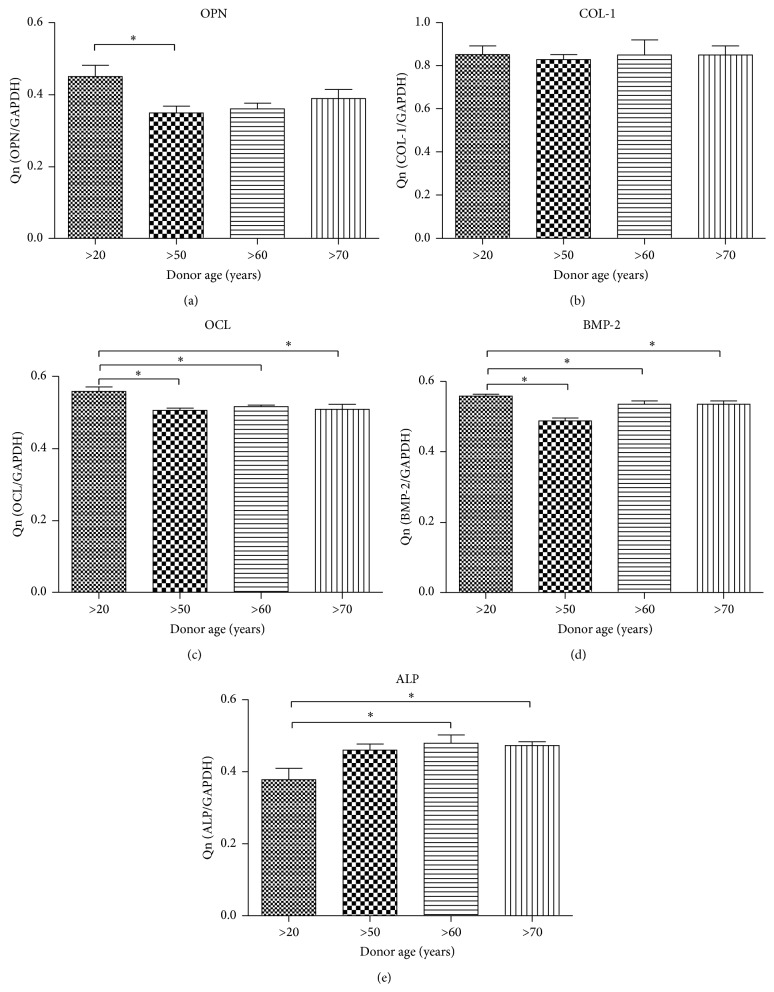
Reverse transcriptase-polymerase chain reaction (RT-PCR) results. Gene expression in hASC cultured in osteogenesis-promoting conditions. Osteopontin (a), collagen type I (b), osteocalcin (c), bone morphogenetic protein 2 (d), and alkaline phosphatase (e) mRNA levels. Results expressed as mean ± SD. ^*∗*^ *P* value < 0.05.

The quantative evaluation of OCL, OPN, BMP-2, and ALP mRNA level reveled significant differences between transcripts expresion between Op derived from youg donors and elderly indyviduals (Figures 8(a)–8(e)). We observed higher expression of OCL, OPN, and BMP-2 mRNA level in younger donors. Interestinlgy, the elevated expresion of ALP mRNA level was noticed in the ederly group when compared with younger donors.

### 3.8. Age-Related Changes in hASC Cultured under Adipogenic Condition

**Figure 9:**
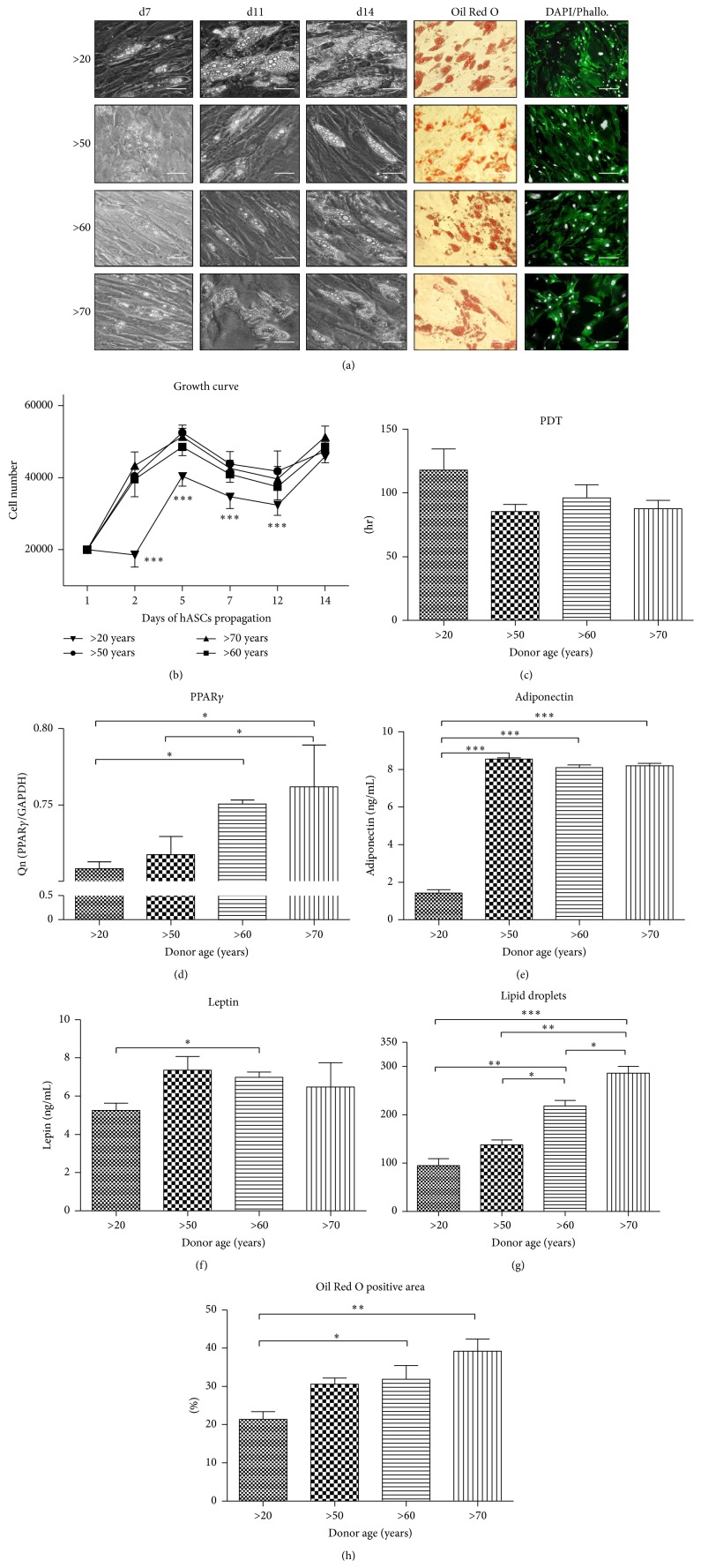
Adipogenic differentation analysis. Morphological evaluation on 7th, 11th, and 14th day. Oil Red O and DAPI/Phalloidin staining performed on 14th day (a); magnification ×100, scale bars: 77 *μ*m; Oil Red O and DAPI/Phallo. Magnification ×100, scale bars: 250 *μ*m. Growth curve (b) showing significantly slower proliferation rate in young patients (>20). No statistically important differences in population doubling time were seen between the different age groups (c). Expression level of PPAR*γ* (d) and concentration of leptin (e) and adiponectin (f) in the supernatants on the 14th day, measured with ELISA assay. Obtained data display differences in leptin amount between >20 and >60 patients, respectively, as well as age-related increase in adiponectin expression in hASC obtained from older subjects (50–70 years). Quantification od adipogenesis using ImageJ software: lipid droplets number (g) and Oil Red O positive area (h). Results expressed as mean ± SD. ^*∗*^ *P* value < 0.05, ^*∗∗∗*^ *P* value < 0.001. (>20), (>50), (>60), and (>70): donor age range, d7: day 7th, d11: day 11th, and d14: day 14th.

To confirm functionality of hASC adipogenic differentiation, adipocytes precursors (Ap) were stained with Oil Red O dye on the 14th day (Figure 9(a)). Formation of first lipid droplets was observed on the 7th day of culture, with the greatest amount of fat droplets in the >20 years group. On the 14th day of culture adipocytes presented comparable amounts of lipid droplets.

Growth kinetics of Ap was evaluated during 14-day culture (Figure 9(b)). The performed test revealed that the Ap derived from elderly donors proliferated significantly faster than Ap of young donors. However, after the 14th day of culture, Ap reached comparable number in all investigated groups. The highest PDT was observed in the >20 years group (118 ± 16.55 h) (Figure 9(c)).

Expression of PPAR*γ* (Figure 9(d)) was significantly higher in age groups between 50 and 70 years old in comparison to young donors (>20). Significantly lower protein concentration of both adiponectin and leptin was observed in younger donors (Figures 9(e) and 9(f)).

Number of lipid droplets (Figure 9(g)) and Oil Red O stained area (Figure 9(h)), was progressively increasing with donor age, showing significant differences between investigated groups.

### 3.9. Analysis of the Oxidative Stress Factors in Ap

**Figure 10:**
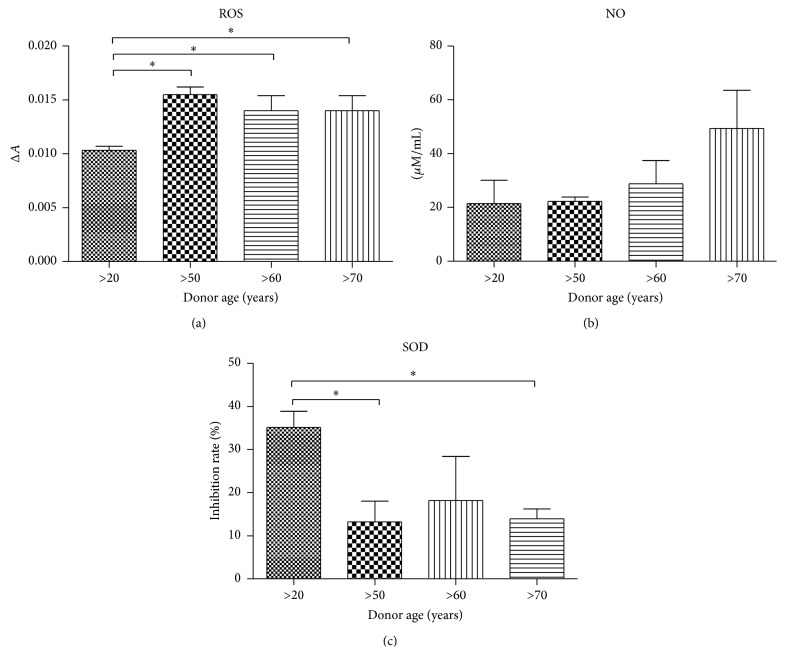
Analysis of oxidative stress factors in adipogenic precursors (Ap). Reactive oxygen species (a), nitric oxide (b), and superoxide dismutase (c) levels. Results expressed as mean ± SD. ^*∗*^ *P* value < 0.05.

ROS activity in Ap culture was significantly lower (*P* < 0.05) in young donors when compared to other age groups (Figure 10(a)). NO concentration differences were not significant between the analyzed groups (Figure 10(b)). Superoxide activity significantly decreased with donor age; it was 2.6-fold lower in the >70 years group when compared with the >20 years group.

## 4. Discussion

Nowadays, rapid ageing of populations becomes a real challenge for national healthcare systems, global economy, and medicine [25]. As the World Health Organization (WHO) predicts, the next few decades will bring a change to the prevalence of diseases making age-related illnesses (e.g., cardiovascular disease, Alzheimer's disease, osteoporosis, arthritis, and diabetes) more common than infectious diseases, childhood diseases, or accidents [26]. Regenerative medicine, including stem cell therapy, may be a potential solution to this problem. Thus, the evaluation of correlation between osteogenic and adipogenic differentiation potential of MSCs, their proliferative activity, viability, and senescence level in the context of their potential clinical applications seems to be justified. Moreover, correlative analysis of oxidative stress factors including ROS, NO, and superoxide dismutase, which is an antioxidant scavenger that protects tissues within the body from oxidative damage [27], might deliver valuable knowledge concerning suitability of MSCs isolated from elderly patients in clinical practice. What is more, obtained data might suggest the necessity to find an alternative approach to elderly donors, which are qualified to stem cell therapy, and might convince clinicians to enhance proliferative and differentiating potential of isolated MSCs, before clinical application.

Isolated hASCs from both younger and older donors exhibited typical for MSCs shape, morphology,and phenotype. Obtained hASCs expressed all of mesenchymal markers, for example, CD44, CD73, CD90, and CD105 and lacked hematopoietic markers: CD34 and CD45. We observed quantitative changes between expressions of CD73 surface marker in relation to donor age, which may contribute to decreased proliferation potential of aged hASCs.

In this study we presented that proliferative activity (PA), clonogenic potential, and PDT were significantly different between hASCs isolated from young patients (>20 years of age) when compared to older donors (50–70 years of age). Interestingly, there were no significant changes in regard to PA and PDT between patients differing in age, what suggests that the proliferative potential of hASCs within the tissue is not correlated with increasing age. Obtained data stand in good agreement with the findings of Zhu et al. [28], who also noticed substantial differences in both PA and PDT, in young patients compared to older donors. Schipper et al. [29] have also observed that hASCs isolated from younger patients proliferate faster when compared with older donors. We have also observed reduced potential of CFU forming in older patients. Decreased proliferation capacity as well as number of CFU, which correlate with hASCs expansion* in vitro*, may be especially questionable when considering usage of those cells in therapies.

The proliferative potential, senescence, and apoptosis level in hASCs isolated from older patients might be directly linked to oxidative stress. In this study we observed a significant tendency to form apoptotic bodies (caspase-3 positive cells) as well increasing number of senescent cells accumulating *β*-galactosidase in regard to patient age, with simultaneously elevated levels of ROS and NO. At the same time we noticed decreased antioxidative protection originating from SOD activity in the group of older donors. This correlates with our observations regarding decreasing mitochondrial activity as well as Golgi bodies in older donors. Obtained data stand in good agreement with Stolzing et al. [30] findings that showed correlation between oxidative stress and apoptosis, as well as senescence level in MSCs culture. It is worth underlining that there were no statistical differences between ROS and NO level in the group of older donors what might indicate that oxidative processes in regard to elderly age are maintained on a comparable level. We also observed, in accordance with Zhou et al. [31], age-related changes in mRNA level of the p53 protein, which is well known for its role in apoptosis induction. Obtained results suggest that upregulation of p53 may be responsible for age-related decreases in proliferation potential, as well as for apoptotic susceptibility of hASCs. We have also observed enlarged nuclei, which is associated with cell senescence [32].

In recent years, many studies indicate the multilineage differentiation potential of hASCs [33]. However, we were interested whether ASCs isolated from older donors might be useful in regenerative medicine and tissue engineering. In the current study, we observed an increase in calcium accumulation, osteo-nodules formation, and elevated expression of BMP-2, OPN, and OCL on mRNA and protein levels in younger donors when compared to the elderly. The observed lower but comparable level of above mentioned gene expression in older donors might suggest to some extent that MSCs show differentiation stability in those patients. Surprisingly there were no statistical differences between expression of COL-1 on the mRNA level between younger and older patients, what might indicate that age dos not effect COL-1 expression on the mRNA level. In addition, we have found a decrease of ROS with simultaneous elevated level of SOD in younger donors when compared to older donors. As reported by Chen and colleagues [34] this might be due to a change in energy production from glycolysis to oxidative phosphorylation in MSCs upon osteogenic induction. In turn, in older donors we observed a linear inverse relation between ROS accumulation and SOD activity. It might suggest that MSCs lose their antioxidative protection with age. Interestingly, we have found that the highest level of NO was detected in the youngest donors in relation to other groups. The obtained data stand in good agreement with Xiao et al. [35] who found that releasing of NO by MSCs, cultured in osteogenic conditioned media, promotes their ability to differentiate into osteoblasts. In turn, Geula et al. [36] have reported that NO also promotes MSCs differentiation into adipocytes. In our study we have observed an elevated level of NO in the oldest group of donors, with simultaneous inhibition of NO secretion in the youngest one. In addition we observed that PF of differentiated MSCs was significantly higher in older donors when compared to young patients. Also, the cultured adipogenic precursors were characterized by significantly shorter PDT in comparison to younger donors. Moreover, we have found that both leptin and adiponectin concentrations were elevated in older donors when compared with younger patients. The simultaneous increase in the level of adiponectin and leptin in differentiated MSCs derived from older patients might be to some extent considered as a factor negatively effecting bone regeneration. Adiponectin and its receptor are expressed in osteoblasts; thus this substance may play an important role in bone metabolism. As studies show, serum adiponectin level is negatively correlated with bone mineral density [37]. Some authors also postulate the theory of the so-called “adipogenic switch.” It states that in advanced age MSCs lose their osteogenic potential and in turn gain an adipogenic potential [38], which leads to senile osteoporosis [39]. This correlates well with our results, as we have observed decreased BMP-2 gene expression in older patients, which might support the mentioned theory, as this cytokine is essential in osteoblasts maturation. We have also noticed increased expression of PPAR*γ* in elderly patients. This protein is known as a central transcriptional regulator of adipogenic pathway. Expression of PPAR*γ* in ASCs increases with age; thus it may be a major factor controlling cells fate by activating adipogenesis at the expense of osteogenesis [40]. Thus it is possible that increased number of adipocytes and decreased number of osteoblast in differentiated ASCs may be due to upregulated PPAR*γ* activity. This imbalance in differentiation capacity results in reduced bone formation and increased risk for osteoporosis [30]. Moreover, we found an increased level of ROS in adipogenic differentiated MSCs with simultaneous lipid accumulations in the group of older donors, which may suggest that ROS accumulation does not decrease adipogenic potential.

Aging is a complex process, at which molecular basis still remains to be elucidated. Epigenetic changes like histones modification and DNA methylation are thought to play a significant role in this process [41]. It is known that in many aged cell types the global DNA methylation status is significantly increased [42]. Recent studies showed that 5-Azacytidine, which inhibits the activity of DNA methyltransferases (DNMTs) promotes DNA demethylation in aged hASCs. Moreover it increased cell proliferation and accelerated osteogenic differentiation, causing rejuvenation of aged cells [43]. Another approach to hASC rejuvenation may be treating them with antioxidants to reduce ROS levels. A study conducted by Lin et al. [44] revealed that culturing hASCs in a growth medium with a low concentration of calcium and supplemented with N-acetyl-L-cysteine, as well as L-ascorbic acid-2-phosphate not only accelerated stem cells growth but also prolonged their lifespan.

## 5. Conclusions

The current study revealed that growth kinetics of hASCs is positively correlated with donor age. The number of both apoptotic and senescent cells increases with age. While hASCss osteogenic differentiation potential decreased with donor age, the adipogenic differentiation potential seemed to remain on the same level throughout the whole ageing process. Demonstrated results show the need for efficient biotechnological solutions that might rejuvenate MSCs* in vitro*, especially when clinical applications are considered.

## Figures and Tables

**Table 1:** Sequences of primers used in qPCR. PPAR*γ*: peroxisome proliferator-activated receptor *gamma*; COL-1: collagen type I; OPN: osteopontin; BMP-2: bone morphogenetic protein 2; ALP: alkaline phosphatase; OCN: osteocalcin; p53: tumor suppressor p53; GAPDH: glyceraldehyde 3-phosphate dehydrogenase; bp: base pair.

Gene	Primer	Sequence 5′-3′	Amplicon length (bp)	Accession number
PPAR*γ*	F:	AGTCCTCACAGCTGTTTGCCAAGC	125	XM_011533844.1
	R:	GAGCGGGTGAAGACTCATGTCTGTC		

COL-1	F:	GTGATGCTGGTCCTGTTGGT	123	NM_000088.3
	R:	CACCATCGTGAGCCTTCTCT		

OPN	F:	AAACGCCGACCAAGGTACAG	213	U20758.1
	R:	ATGCCTAGGAGGCAAAAGCAA		

BMP-2	F:	ATGGATTCGTGGTGGAAGTG	349	KC294426.1
	R:	GTGGAGTTCAGATGATCAGC		

ALP	F:	CGCGCTTGTGCCTGGA	185	XM_006710546
	R:	CCTGCTTTATCCCTGGAGCC		

OCN	F:	ATGAGAGCCCTCACACTCCTC	292	NM_199173.4
	R:	CGTAGAAGCGCCGATAGGC		

p53	F:	AGATAGCGATGGTCTGGC	381	NM_001126118.1
	R:	TTGGGCAGTGCTCGCTTAGT		

GAPDH	F:	CATGGCCTTCCGTGTTCCTA	286	NM_017008.4
	R:	CACCACCCTGTTGCTGTAGC		
